# Is it safe to have multiple repeat cesarean sections? A high volume tertiary care center experience

**DOI:** 10.12669/pjms.335.12899

**Published:** 2017

**Authors:** Alper Biler, Atalay Ekin, Aykut Ozcan, Abdurrahman Hamdi Inan, Tayfun Vural, Emrah Toz

**Affiliations:** 1 Alper Biler, Department of Obstetrics and Gynecology, Tepecik Training and Research Hospital, Izmir, Turkey; 2 Atalay Ekin, Department of Obstetrics and Gynecology, Tepecik Training and Research Hospital, Izmir, Turkey; 3 Aykut Ozcan, Department of Obstetrics and Gynecology, Tepecik Training and Research Hospital, Izmir, Turkey; 4 Abdurrahman Hamdi Inan, Department of Obstetrics and Gynecology, Tepecik Training and Research Hospital, Izmir, Turkey; 5 Tayfun Vural, Department of Obstetrics and Gynecology, Tepecik Training and Research Hospital, Izmir, Turkey; 6 Emrah Toz, Department of Obstetrics and Gynecology, Tepecik Training and Research Hospital, Izmir, Turkey

**Keywords:** Complication, Morbidity, Multiple repeat cesarean section

## Abstract

**Objective:**

To compare the obstetric outcomes of cesarean section in women who had a history of four or more previous cesarean sections with those who had a history of two or three previous cesarean sections.

**Methods:**

Total 1318 women who underwent repeat cesarean section between January 2013 and January 2016 were retrospectively reviewed. Of these, 244 (18.5%) had previously had four or more cesarean sections (multiple repeat cesarean section group) and 1074 (81.5%) had previously had two or three cesarean sections (control group). Demographic characteristics and obstetric outcomes were compared using the Independent t and chi-square tests.

**Results:**

The adhesion rate (p < 0.001), number of blood transfusion (p = 0.044), operation time (p = 0.012), length of hospital stay (p < 0.001) and tubal ligation surgery (p < 0.001) were significantly higher in multiple repeat cesarean section group compared to control group.

**Conclusion:**

Although multiple repeat cesarean section are asscociated with adhesion occurrence, higher number of blood transfusion, increased operation time and length of hospital stay, there is no remarkable difference in serious morbidity associated with multiple repeat cesarean section.

## INTRODUCTION

Cesarean deliveries have risen significantly over the past decades due to advanced maternal age, defensive obstetric practice, medicolegal concerns and maternal request. In Turkey, the cesarean section (CS) rate increased from 8% to 37% between 1993 and 2008.^1^ CS is a surgical procedure including some risks such as uterine rupture, infection, hemorrhage, thrombosis and damage to the bladder, ureters or bowel.^2^ Although CS is now safe along with devolopments in anesthesia and surgery, these complications of CS can be life-threatening for both mother and baby.^3^ Compared with primary CS, multiple repeat caesarean sections (MRCS) are associated with additional risks including placenta previa, abnormal placental invasion and difficulties in surgical dissection.

Due to the overall rise in cesarean frequency in developing countries, an increasing number of women have had MRCS. Trial of labor after CS is an alternative to decrease CS rates. However, vaginal birth after CS is not being routinely performed in all hospitals of our country. Furthermore, many clinicians suggest sterilization to women following two or three CSs due to risk of uterine rupture and several complications. However, most women do not accept sterilization in Turkey where large families are encouraged for social and cultural reasons. In addition, there has been an ongoing debate about the recommended maximum number of CSs that a woman may safely have.

The aim of this study was to compare the obstetric outcomes of CS in women who had a history of four or more previous CSs with those who had a history of two or three previous CSs.

## METHODS

The study was performed by a retrospective evaluation of the hospital records at the Obstetrics and Gynecology Department of Tepecik Training and Research Hospital, Izmir, Turkey, between January 2013 and January 2016. This study was performed in accordance with the ethical standards of the Declaration of Helsinki and was approved by the local ethics committee at our institution. During the study period, total number of deliveries were 58669. A total of 28032 pregnant women who had undergone CS were investigated from the database. Women with multiple pregnancies and prior classic, T, or low vertical incisions were not included. Among all CSs, 26714 women were excluded from the analyses due to first CS (n=25196), multiple pregnancies (n = 1219), incomplete data (n = 248) and incision type (n = 51). The study group, thus, comprised 244 women who had undergone four or more CS and was called as MRSC group. The control group was comprised 1074 women who had undergone two or three CSs. Demographic characteristics and maternal outcomes were compared between two groups.

Following demographic parameters of study population were collected: maternal age, gravidity, parity, number of previous CSs, gestational week at delivery, presence of additional diseases, birthweight, Apgar score at 5 min and tubal ligation surgery. Additional diseases were systemic illnesses such as hypertension, preeclampsia, diabetes and chronic renal disease. Intraoperative and postoperative parameters included presence of adhesion, operation time, length of hospital stay, preoperative and postoperative hemoglobin (Hb) levels, blood transfusion, placenta previa, abnormal placental invasion, uterine rupture, cesarean hysterectomy, bladder and bowel injury and maternal death.

In our hospital, elective CSs were performed at 39 gestational weeks after confirming gestational age by first trimester crown rump length measurement. Emergency CS was performed in the setting of non-reassuring fetal status, failure to progress in labor or labor after previous CS. All operations were performed by a specialist obstetrician. In general, a Pfannenstiel skin incision was made and carried down through layers to open the abdominal cavity. The uterus was opened with a transverse lower segment incision. The duration of the operation was calculated from the time between initiation of anesthesia and closure of the skin incision. Length of hospital stay indicated the time between the completion of CS and hospital discharge. Blood transfusion was given if preoperative Hb was < 8 g/dl, or when the anticipated surgical blood loss exceeded 1000 ml. The severity of the pelvic adhesions was graded according to American Fertility Society Classification of adnexal adhesions.^4^ Adhesions involving 1% to 25% of the total area are classed as mild, between 26% and 50% of pelvic area is moderate and greater than 50% of the area are severe. Placenta previa was defined as the placental implantation over the internal cervical os or within 2 cm of it. We used the term abnormal placental invasion for placenta accreta, increta and percreta. Placental invasion abnormalities were defined according to surgery reports by the surgeon during surgery as a difficult manual removal with no cleavage plane identified between the placenta and uterus, resulting in incomplete removal or need to leave the entire placenta in situ and invasion of other pelvic organs. Pathological confirmation was not routinely used. Uterine rupture was defined as full-thickness separation of a prior uterine scar.

Statistical analysis was performed using SPSS (Statistical Package for the Social Sciences for Windows version 20.0, SPSS Inc., Chicago, Illinois, USA). Data were expressed as mean ± standard deviation for continuous variables and number of cases and percentages for categorical variables. Continuous parametric variables between groups were compared by Independent t-test. Categorical data was analyzed by chi-square test. A p-value < 0.05 indicated statistical significance.

## RESULTS

Of the 244 patients in MRCS group, 217 patients had four previous CSs, 26 had five and 1 had seven previous CSs. Of the 1074 patients in control group, 387 had two previous CS and 687 had three previous CSs.

The demographic characteristics of the two groups are presented in Table-I. Maternal age (32 ± 4.6 y vs. 29.6 ± 5.4 y, p < 0.001), gravidity (4.7 ± 1.1 vs. 2.9 ± 1.9, p < 0.001), parity (3.2 ± 0.6 vs. 1.4 ± 0.5, p< 0.001), presence of additional disease (53.6% vs. 12.5%, p < 0.001) and tubal ligation (56.1% vs. 21.9%, p < 0.001) are statistically significantly higher in MRSC group when compared to control group. The gestational age at delivery (37.9 ± 1.6 wk vs. 38.1 ± 1.8 wk, p = 0.175), mean birth weight (3173.8 ± 556.6 g vs. 3143.3 ± 1160.6 g, p = 0.689) and Apgar score at 5 minutes (7 ± 0.8 vs. 6.8 ± 0.9, p = 0.068) were not significantly different between groups.

**Table-I:** The demographic characteristics of the study population.

*Variable*	*MRCS group (n = 244)*	*Control group(n = 1074)*	*p*
Maternal age (y)	32 ± 4.6	29.6 ± 5.4	< 0.001
Gravidity	4.7 ± 1.1	2.9 ± 1.9	< 0.001
Parity	3.2 ± 0.6	1.4 ± 0.5	< 0.001
Gestational age at delivery (wk)	37.9 ± 1.6	38.1 ± 1.8	0.175
Birthweight (g)	3173.8 ± 556.6	3143.3 ± 1160.6	0.689
Apgar score at 5 min	7 ± 0.8	6.8 ± 0.9	0.068
Additional disease	131 (53.6%)	135 (12.5%)	< 0.001
Tubal ligation	137 (56.1%)	236 (21.9%)	< 0.001

Values are presented as mean [SD] or n (%) unless otherwise specified. MRCS, multiple repeat cesarean section

Maternal outcomes according to groups were shown in Table-II. Compared to control group, the patients in MRSC group had significantly higher adhesion incidence (46.7% vs. 23.8%, p < 0.001) and number of blood transfusion (0.3 vs. 0.1, p = 0.044). Furthermore, operation time (44.3 ±5.3 min vs. 40.9 ± 17.9 min, p = 0.012) and length of hospital stay (60 ± 40.9 h vs. 52.2 ± 20.8 h, < 0.001) were significanty longer in MRSC group compared to control group. There were not significant differences among two groups in terms of preoperative (11.4 ± 1.3 (g/dL) vs. 11.5 ± 1.3 (g/dL), p = 0.105) and postoperative Hb (9.9 ± 1.4 (g/dL) vs. 10.2 ± 2.8 (g/dL), p = 0.089), Hb decrease (1.5 ± 1.1 (g/dL) vs. 1.3 ± 2.6 (g/dL), p = 0.359), blood transfusion (8.6% vs. 6%, p = 0.145), placenta previa (4% vs. 2.7%, p = 0.245), abnormal palcental invasion (4.5% vs. 3%, p = 0.26), uterine rupture (0.8% vs. 0.4%, p = 0.492), cesarean hysterectomy (1.2% vs. 0.9%, p = 0.67), bladder (0.4% vs. 0.3%, p = 0.932) and bowel injury (0 vs. 0). All patients with uterine rupture were treated successfully. One patient in the MRCS group required cesarean hystrectomy. There was no maternal death in both groups.

**Table-II:** Comparison of maternal outcomes according to MRSC and control groups.

	*MRSC Group (n = 244)*	*Control Group (n = 1074)*	*p*
Adhesion	114 (46.7%)	256 (23.8%)	< 0.001
Mild	57	187	0.03
Moderate	32	41	< 0.001
Severe	25	28	< 0.001
Operation time (min)	44.3 ± 25.3	40.9 ± 17.3	0.012
Length of hospital stay (h)	60 ± 40.9	52.2 ± 20.8	< 0.001
Preop Hb (g/dL)	11.4 ± 1.3	11.5 ± 1.3	0.105
Postop Hb (g/dL)	9.9 ± 1.4	10.2 ± 2.8	0.089
Hb decrease (g/dL)	1.5 ± 1.1	1.3 ± 2.6	0.359
Blood Transfusion	21 (8.6%)	65 (6%)	0.145
Number of blood transfusion	0.3 ± 1.3	0.1 ± 0.9	0.044
Plasenta previa	10 (4%)	29 (2.7%)	0.245
Abnormal placental invasion	11 (4.5%)	33 (3%)	0.26
Uterine rupture	2 (0.8%)	5 (0.4%)	0.492
Cesarean hysterectomy	3 (1.2%)	10 (0.9%)	0.67
Bladder injury	1 (0.4%)	4 (0.3%)	0.932
Bowel injury	0	0	
Maternal death	0	0	
Type of anesthesia			0.142
Regional	220 (90.2%)	998 (92.9%)	
General	24 (9.8%)	76 (7.1%)	

Values are presented as mean [SD] or n (%) unless otherwise specified. MRCS, multiple repeat cesarean section.

## DISCUSSION

In the past 5 years, the cesarean delivery rate has substantially increased in our center. The rate was 32.8% of all births in 2011, 44.1% in 2012, 57.7% in 2013, 56.7% in 2014 and 56.6% in 2015. The significant rise in the cesarean delivery rate can be attributed to many factors such as, inadequate knowledge of contraceptive, advanced maternal age, maternal preference and medico-legal concerns. Compared with primary cesarean delivery, repeat cesarean delivery could be associated with additional risks.

In our study, we observed that the adhesion rate of women who had four or more CSs was significantly higher than those who had three or less CSs (46.7% vs. 23.8%). These results are in agreement with those obtained by previous studies.^3^,^5^-^10^ The adhesion rate in the study of Rashid et al. was found to be 54% for women who had five or more CSs and 15% for women who had two to three previous CSs.^3^ The higher incidence of adhesion in MRCS group is mainly resulted from the higher total number of recurrent surgery on the abdominal wall. CSs are often associated with desiccation of peritoneal surfaces, exposure to vaginal flora and residual blood. There is no doubt that every additional CS is at least as morbid as the first one.^3^ It is also possible that adhesions are affected by the surgical technique, gentle tissue management and general health situation of the patient influence tissue healing.^8^

Placental invasion anomalies are one of the life threatining complications of pregnancy with a prevalance of 1/500 to 1/2500 pregnancies. The most important risk factors are previous CS and placenta previa.^11^,^12^ Several reports have shown that the incidence of placental invasion abnormalities increases with increasing number of CS.^2^,^5^,^13^,^14^ In addition to these reports, our study demonstrated that incidence of abnormal placental invasion in patients who had undergone four or more CSs did not significantly different from patients who had undergone two or three CSs. Similarly, Gasim et al. compared 144 pregnant women with ≥ 4 CSs with a control group of 288 women having 2-3 CSs for maternal, operative and neonatal complications.^15^ They showed that there were no significant differences between groups in terms of serious complications including placenta accrete.^15^

Uterine rupture is one of the most catastrophic complications of pregnancy and can also present as an asymptomatic scar dehiscence. It has already known that myometrial thinning usually progressed with the increasing number of previous CSs. In the present study, uterine rupture was observed in 2 patients in the MRCS group and in 5 patients in the control group. Our results demonstrated similar uterine rupture rates in the MRCS group compared with the control group. Juntunen et al. observed that the women with four or more CSs were more commonly encountered with fenestration of the uterine scar.^5^ In this study, all of our patients were treated successfully. There were both placenta previa and abnormal placental invasion in 3 patients. One patient was undergone cesarean hysterectomy in the MRCS group.

Another reported significant morbidity in women with repeated CSs is cesarean hysterectomy. Abnormal placental invasion and uterine rupture are the most common indications for cesarean hysterectomy.^3^ However, our results showed no significant difference in the rates of cesarean hysterectomy between two grops. This result may be explained by the fact that we did not find significant differences in the incidences of uterine rupture and abnormal placental invasion between groups. In connection with these results, the incidence of peripheral organ damage such as bowel and bladder injury was not significantly different among groups. Bladder injury which was managed with a primary suture repair was observed in only 0.4% of patients in MRSC group and 0.3% of patients in control group. Bowel injury was not seen in any of the patients.

The factors which MRSC has been linked with minor morbidity such as operation time, length of hospital stay and number of blood transfusion were compared between two groups. It is not surprising that previous CS was a major risk factor for increased operation time. Consistent with this finding, Rashid et al. found that higher order (5-9) repeat cesarean sections were associated with a longer operating time when compared with lower order (3 or 4) repeat CSs.^3^ The main reason for increased operating time was diffuculties experienced with dissection of the abdominal wall and separation of the bladder from the lower uterine segment due to severe adhesions. The duration of hospital stay was significantly longer in the MRCS group than control group. A possible explanation for this might be the presence of adhesions, long operation time, advanced maternal age and presence of concurrent additional diseases of mothers. As expected, age, gravidity, parity and additional diseases were found to be higher in the MRCS group.

The preoperative and postoperative Hb levels and the incidence of blood transfusion was similar in the two groups. However, the number of blood transfusion was significantly greater in the study group. Similar to our study, Rouse et al. reported that the risk of blood transfusion increased significantly as the number of previous CSs increased.^16^ In that study, blood transfusion rates of women with 1, 2, 3, 4, and at least 5 CSs were found to be 1.8%, 2.6%, 4.3%, 4.6%, and 14.6%, respectively (p < 0.001). Silver et al. observed that the risk of transfusions of ≥ 4 units of red blood cells was associated significantly with an increased number of CSs.^17^ Major reason for excessive hemorrhage after CS was thought to be adhesions.

The results of our study indicate that women who had ≥ 4 CSs have not associated with major risk factors compared with those who had two or three CSs. Prior studies showed that no absolute upper limit for the number of repeat CSs can be given. Cook et al. found that women with five or more CSs had significantly more major obstetric complications than women with lower order repeat CSs.^2^ In contrast, Rashid et al. reported that five or more CSs were not associated with additional risk factors for mother and fetus when compared with four or less CSs.^3^ Similarly, Seidman et al. observed that four or more CSs have a small risk for the mother but may be related with increased neonatal morbidity referred to primarily preterm non-elective CSs.^7^ Our study is limited by the the retrospective nature of the study plan which causes missing data and incomplete gathering of the necesary information. Strength of this study is the large sample size that increases its power.

## CONCLUSIONS

In conclusion, repeated CSs (four or more) do not appear to increase the risk of maternal complications except for the rate of intra-abdominal adhesions. Although there is no remarkable difference in serious morbidity associated with MRSC, it should be kept in mind that CS is a operative delivery including some risks such as uterine rupture, infection, hemorrhage, thrombosis and peripheral organ damage. Further studies are needed to make a recommendation to women on the maximum number of CSs which should be performed.

### Author’s Contribution

**AB** designed, writing, data collection, conceived the study, did editing of manuscript.

**AE** designed, statistical analysis and writing.

**AO and AHI** did data collection.

**TV and ET** did review and final approval of manuscript.

**AB** takes the responsibility and is accountable for all aspects of the work in ensuring that questions related to the accuracy or integrity of any part of the work are appropriately investigated and resolved.
